# Supplementary material for: Electroassisted Incorporation of Ferrocene within Sol–Gel Silica Films to Enhance Electron Transfer
Source: Molecules. 2023 Sep 28;28(19):6845. doi: 10.3390/molecules28196845 (PMC10574706; doi:10.3390/molecules28196845)
Supplement: Supplementary file 1 [file molecules-28-06845-s001.zip › molecules-2608546-supplementary.pdf]

Supporting information for:

Electroassisted incorporation of Ferrocene within Sol-Gel Silica Films to enhance Electron Transfer

Rayane-Ichrak Loughlani, Alonso Gamero-Quijano, Francisco Montilla

Figure S1 shows the stabilised cyclic voltammograms of the ITO electrode immersed in a test solution of ferrocenium hexafluorophosphate ($FcPF_6$) 0.30 mM + Trizma buffer. Either anodic or cathodic peak potentials and intensities were obtained from the cyclic voltammograms recorded at different scan rates (10, 20, 50, 100, 200, 500, 1000 mV s^{-1}).

The diffusion coefficient has been calculated using the Randles-Sevcik equation. The results indicated that the redox reaction of the Fc/ Fc^+ couple was a diffusion-controlled single electron transfer.

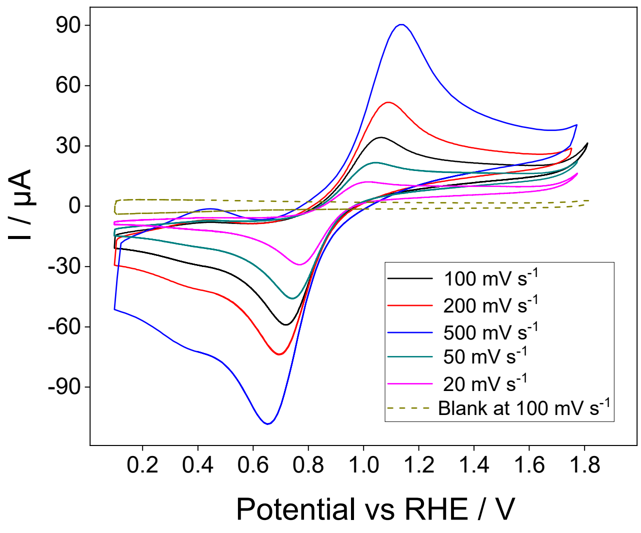

Figure S1. CV at different scan rates of an ITO electrode immersed in an aqueous solution containing ferrocenium hexafluorophosphate ($FcPF_6$) 0.30 mM in Trizma buffer. The dashed line is the blank voltammogram of the ITO electrode of 1.8 cm^2 immersed in trizma buffer solution at (dashed line). Scan rates (SR) are indicated in the figure.

As indicated in Fig S2, the cyclic voltammogram obtained at 10 mV s^{-1} presents an anodic peak with a single bell-shape centered at 1.23 V attributed to the oxidation of neutral ferrocene to ferrocenium. The counter process was observed as a cathodic peak centered at 0.68 V. The peak-to-peak separation was 550 mV, the latter increased around 20 mV by increasing the scan rate to 20 mV s^{-1}. Increasing the scan rates, a pre-peak appears at 1 V changing the anodic peak shape features. This process becomes more evident at higher scan rates (50 mV s^{-1} -1000 mV s^{-1}).

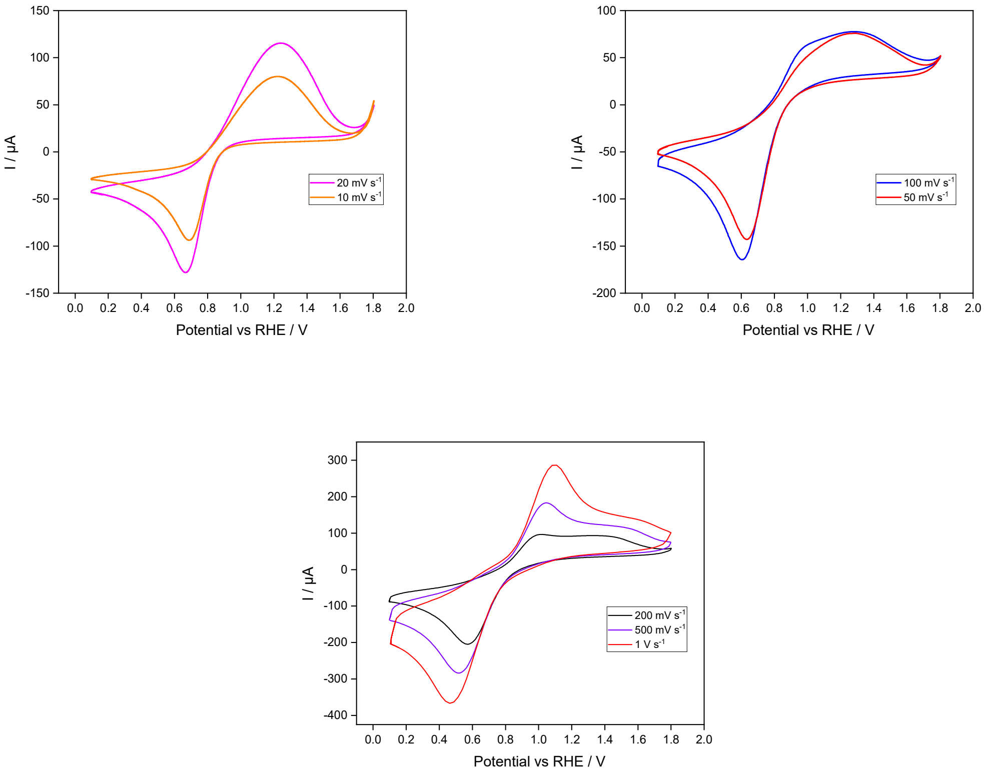

Figure S2. Cyclic voltammograms were obtained for an ITO/SiO_2 modified electrode in an aqueous solution containing ferrocenium hexafluorophosphate ($FcPF_6$) 0.36 mM + Trizma aqueous solutions. Recorded at different scan rates: (10, 20, 50, 100, 200, 500, 1000 mV s^{-1}).

Figure S3 shows the N_2 adsorption isotherms and the pore width distribution for a SiO_2 sample. According to the IUPAC classification, the adsorption isotherm is type IV [1,2]. The microporous volumes obtained from N_2 adsorption indicate that the material has a micropore size. The plot of pore width distribution for our SiO_2 sample indicates the presence of micropores and mesopores. The particulate property of the simple such as surface area, pore size, and volume distribution, was listed in the table.

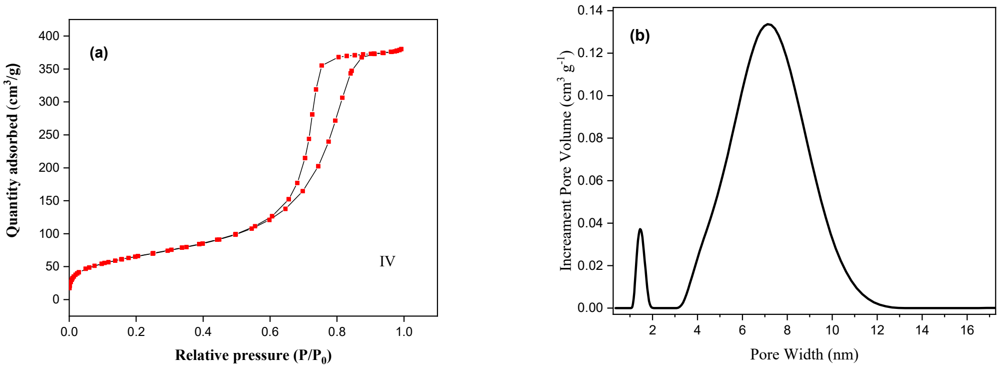

Figure S3. (a) N_2 adsorption isotherms at 77.35 K for a SiO_2 sample. (b) Pore width distribution for a SiO_2 sample.

Table S1. Textural properties of the SiO_2 sample obtained from the N_2 adsorption isotherms.

Sample	S_{BET} (m^2 g^{-1})	V N_2 (cm^3 g^{-1})	Ads P_D (nm)	Des P_D (nm)
SiO_2	237.26	0.58	5.28	6.94

Figure S4 shows repetitive cyclic voltammetry of an ITO electrode immersed in 0.24 mM (Fig. S4 A) and 0.27 mM (Fig. S4 B) of ferrocenium hexafluorophosphate ($FcPF_6$) + Trizma buffer solution. It is worth mentioning that in 0.24 mM, a couple of anodic peaks at 1 V and 1.45 V were evident, but in the reverse scan, we recorded a single cathodic peak centered at 0.57 V. The redox peak current centered at 1.45 V disappeared after repetitive CV cycling (ca. 7 consecutive scans). The appearance of a second peak also leads to a decrease in the reduction current with cycling time and might be related to electrode fouling. This behavior becomes more noticeable, increasing the concentration to 0.27 mM.

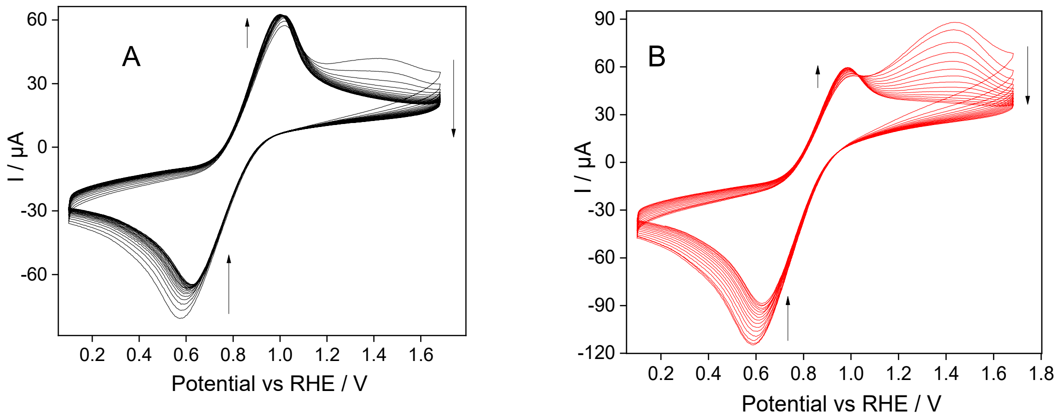

Figure S4. repetitive cyclic voltammetry of an ITO electrode immersed in 0.24 mM (A) and 0.27 mM (B) of ferrocenium hexafluorophosphate ($FcPF_6$) + Trizma buffered aqueous solution. Scan rate: 100 mV s^{-1}.

Figure S5A shows the spectral absorption curve in the visible region of ITO/SiO_2 (dashed line) modified electrode prepared as indicated in the experimental section and ITO/Fc@SiO_2(solid line) modified electrode prepared as indicated in section 2.2. and 2.3 in the main manuscript and dried for one day at room temperature. The recorded UV-vis spectrum obtained with Fc@SiO_2 modified ITO electrode (solid line) exhibits a single absorption band at 620 nm, which can be attributed to the ligand-to-metal charge transfer band of ferrocenium (Fc^+)[3] located within the silica matrix. These findings strongly suggest the entrapment of only ferrocenium species within the silica matrix. The UV-vis spectrum obtained with silica (the dashed line spectrum), we observe only a broad absorption band from (450-750) with a low absorption intensity that is attributed to the absorption of the silica matrix. Figure S7B shows an actual photograph of a dried ITO/SiO_2 and ITO/Fc@SiO_2 modified electrodes, The existence of ferrocenium species was visually confirmed by the electrode's color change from transparent film to blue film.

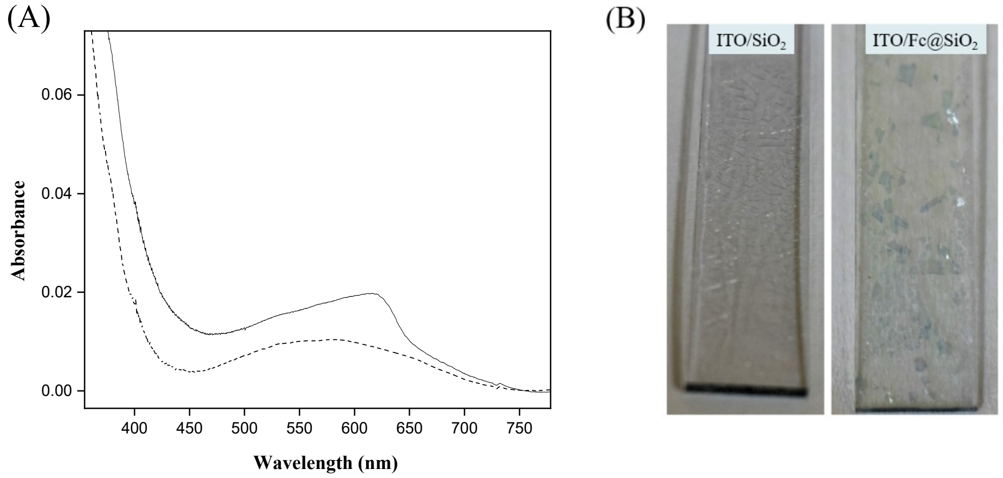

Figure S5. (A) UV−vis spectrum was recorded for SiO_2 and Fc@SiO_2 modified electrode. **(B)** photograph of dried ITO/SiO_2 and ITO/Fc@SiO_2 modified electrodes.

Figure S6 shows the spectral absorption curve in the visible region of ferrocene (solid line) and ferrocenium (dashed line) dissolved in dichloroethane. In the (solid line) absorption spectrum, an absorption band with maximum absorption at 441 nm was observed. This band corresponds to the absorption of ferrocene with a Fc (II) center, and it is attributed to a π−π* transition in the C-rings[4]. The absorption spectrum of ferrocenium species Fc (III) center is shown in dashed line. Ferrocenium presents an absorption band with maximum absorbance centered around 620 nm. This band corresponds to ligand-to-metal charge transfer (LMCT) [3].

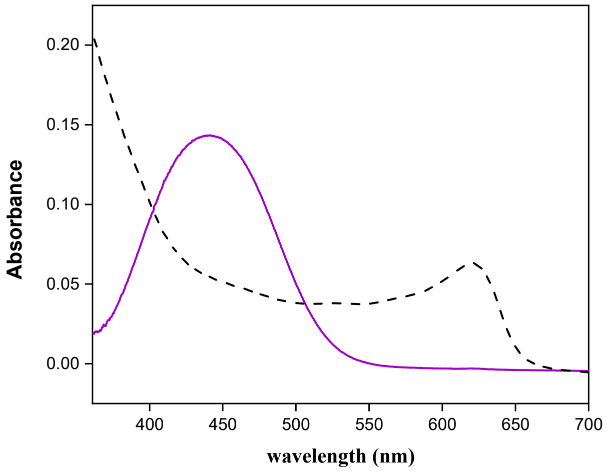

Figure S6. UV−vis spectrum was recorded for ferrocene 0.9 mM (solid line) and ferrocenium hexafluorophosphate ($FcPF_6$) 0.3 mM (dashed line) dissolved in dichloroethane.

Figure S7 displays the XPS spectra obtained for ITO/SiO_2 and ITO/Fc@SiO_2 modified electrode. We can observe several notable features: a peak at a binding energy of 103 eV that is attributed to the silicon oxides/silicon[5] (Si2p). Another prominent peak at 283 eV that is attributed to the silicon carbide(C1s)[6]. We observe a hight intensity peak at 535 eV that corresponds to the silicon oxides/silicon (O1s)[7].

It is noteworthy the absence of iron signal in the Fc@SiO_2 sample (fig. S9F), it is essential to consider the surface sensitivity of XPS. Typically, the $Fe2p_{3/2}$ component is found within the binding energy (BE) range of (709-711) eV[8], as per the existing literature. However, given XPS's surface-sensitive nature, the lack of iron in the Fc@SiO_2 sample may suggest that the accumulated iron is confined within the inner layers of the dried silica films.

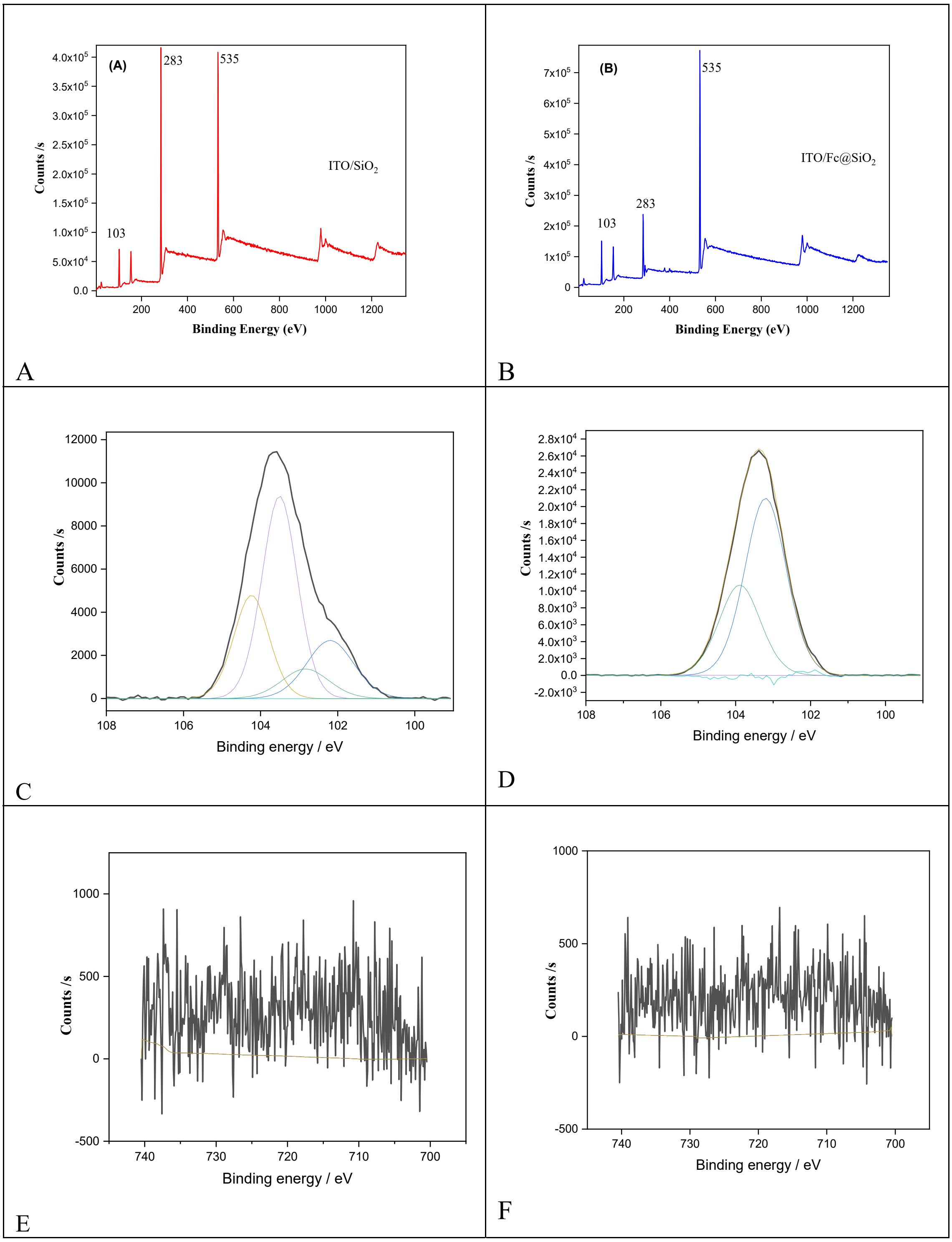

Figure S7. The XPS spectra obtained for (A) ITO/SiO_2 and (B) ITO/Fc@SiO_2 modified electrode. High resolution XPS spectra of (C) ITO/SiO_2 and (D) ITO/Fc@SiO_2 corresponding to Si2p signals. High resolution XPS spectra of (E) ITO/SiO_2 and (F) ITO/Fc@SiO_2 corresponding to Fe2p signals.

Figure S8 shows the evolution of the absorbance vs the ferrocenium species concentrations. The curve shows a linear relationship, indicating that the absorbance of the ferrocenium (Fc^+) species was directly proportional to its concentration within the tested range. The molar absorption coefficient (ε) of the ferrocenium aqueous solution was 182.04 M^{-1} cm^{-1}; and was estimated from the slope of the calibration curve shown in Fig. S8. This molar absorption coefficient (ε) was used to estimate the concentration of the ferrocenium species confined within the silica films in our *in-situ* UV-vis experiments.

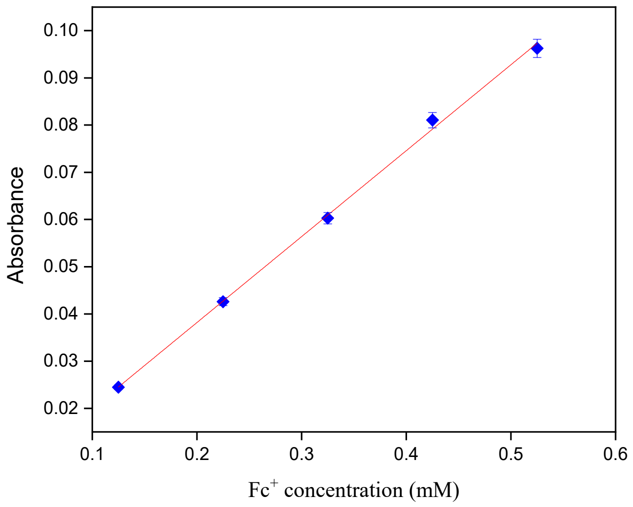

Figure S8. Calibration curve absorbance vs [Fc^+] mM.

Figure S9 shows the stabilised cyclic voltammogram of SiO_2-modified electrode in 1 mg mL^{-1} Cyt c in PBS buffer aqueous solution. The anodic and cathodic waves centred at 1.0 V and 0.64 V were attributed to the oxidation and reduction of cyt c - Fe (II)/Fe (III) species, respectively. The poor cyt c's electrochemical response with SiO_2-modified electrodes could be explained by strong electrostatic interactions between the negatively charged silica and the protein. The isoelectric point of cyt c is around 10 [9]; thus, at neutral pH, cyt c will present a positive net charge. These electrostatic interactions determine the cyt c orientation within the porous silica matrices and probably hinder the charge transfer processes. It is worth mentioning that cyt c presents a diameter of 3 nm; therefore, it will have limited access through the silica pores.

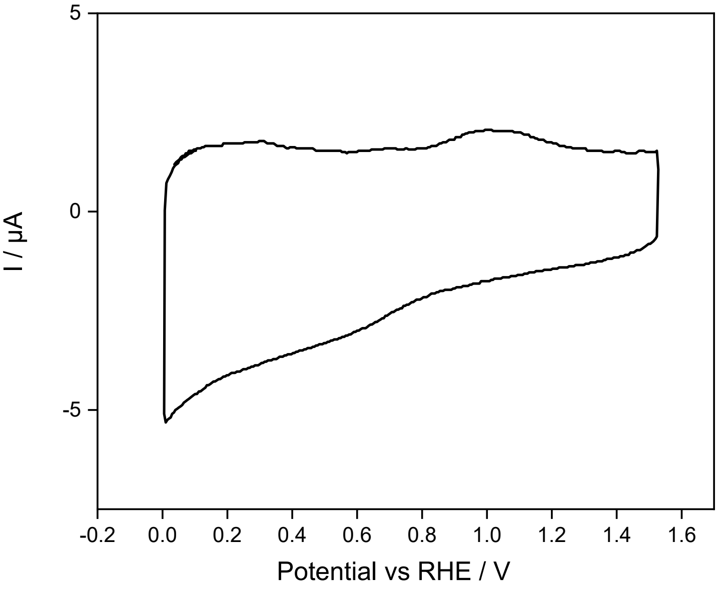

Figure S9. Stabilised cyclic voltammograms of SiO_2-modified electrode in 1 mg mL^{-1} Cyt c in PBS buffer aqueous solution. Scan rate: 100 mV s^{-1}.

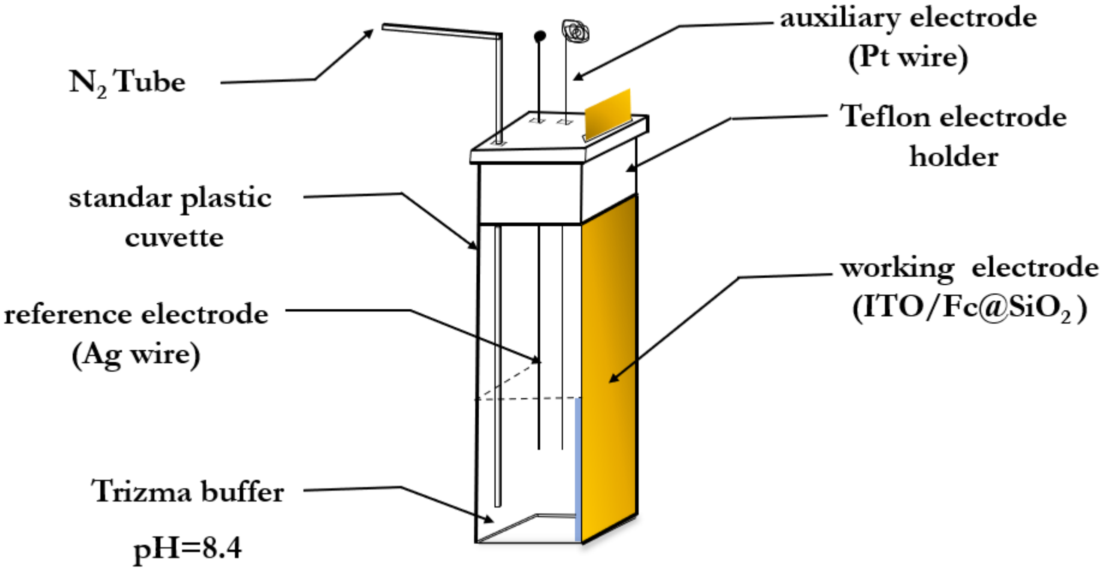

Figure S10: In-situ UV-vis setup. The ITO-SiO_2 modified electrode was held at a constant potential of 1.24 V for six minutes before recording the oxidation spectrum. Similarly, a constant reduction potential of 0.2V was applied for six minutes before taking the reduction spectrum.

The scheme in Figure S2 shows the Sol-Gel silica synthesis. The latter was carried out in two stages, The first stage (see part (1) in Figure S2), was the hydrolysis of the tetraethyl orthosilicate (TEOS), and the latter is based on a mechanical procedure that consists of mixing TEOS with water in the presence of ethanol as a co-solvent and the addition of the HCl as an acidic catalyst. During the hydrolysis stage (around 1h under magnetic stirring), the viscosity of the solution gradually increases, generating a silica sol (colloidal suspension of small particles).

The second stage involves the condensation of the resulting silanols, which includes the formation of ≡Si–O–Si≡ bonds (siloxanes). This condensation is achieved by raising the pH of the silica sol and directly modifying the ITO electrode using the synthesized silica hydrogel matrix (See part (2) in Figure S2). To accomplish this, we combined a Trizma aqueous solution at pH 8.4 with the silica sol in a 1:1 volume ratio in an Eppendorf tube. Subsequently, 40 µL of this mixture was applied to cover an area of 1.8 cm^2 on the ITO electrode. Within 2-3 minutes, a rigid and transparent structure was formed on the ITO electrode.

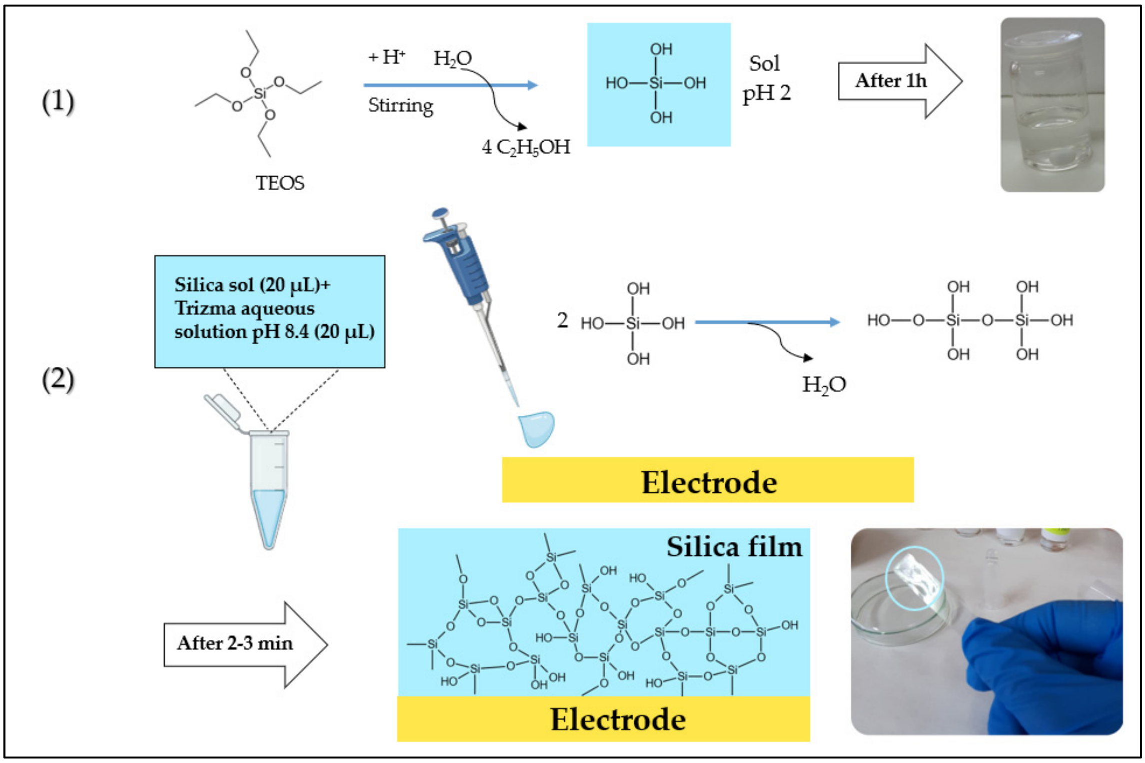

Figure S11: Scheme of the Sol-Gel silica synthesis and modification of the ITO electrodes.

References

(1) Kajama, M. N. Hydrogen Permeation Using Nanostructured Silica Membranes. *Sustain. Dev. Plan. VII* **2015**, *1* (May 2015), 447–456. https://doi.org/10.2495/sdp150381.

(2) Choma, J.; Kloske, M.; Jaroniec, M. An Improved Methodology for Adsorption Characterization of Unmodified and Modified Silica Gels. *J. Colloid Interface Sci.* **2003**, *266* (1), 168–174. https://doi.org/10.1016/S0021-9797(03)00573-3.

(3) Fery-Forgues, S.; Delavaux-Nicot, B. Ferrocene and Ferrocenyl Derivatives in Luminescent Systems. *J. Photochem. Photobiol. A Chem.* **2000**, *132* (3), 137–159. https://doi.org/10.1016/S1010-6030(00)00213-6.

(4) Scott, D. R.; Becker, R. S. Comprehensive Investigation of the Electronic Spectroscopy and Theoretical Treatments of Ferrocene and Nickelocene. *J. Chem. Phys.* **1961**, *35* (2), 516–531. https://doi.org/10.1063/1.1731962.

(5) Cardinaud, C.; Rhounna, A.; Turban, G.; Grolleau, B. Analyse XPS Des Surfaces de Si et SiO2 Exposées Aux Plasmas de CHF3 et CHF3—C2F6. Polymérisation et Gravure. *Rev. Phys. appliquée* **1989**, *24* (3), 309–321.

(6) Johansson, L. I.; Owman, F.; Mårtensson, P. High-Resolution Core-Level Study of 6H-SiC (0001). *Phys. Rev. B* **1996**, *53* (20), 13793.

(7) Hollinger, G. Structures Chimique et Electronique de l'interface SiO2-Si. *Appl. Surf. Sci.* **1981**, *8* (3), 318–336.

(8) Paparazzo, E. XPS and Auger Spectroscopy on Mixtures of the Oxides SiO_2, Al_2O_3, Fe_2O_3, and Cr_2O_3. *J. Electron Spectros. Relat. Phenomena* **1987**, *43*, 97–

112.

(9) Hristova, S. H.; Zhivkov, A. M. Isoelectric Point of Free and Adsorbed Cytochrome c Determined by Various Methods. *Colloids Surfaces B Biointerfaces* **2019**, *174* (November 2018), 87–94. https://doi.org/10.1016/j.colsurfb.2018.10.080.
